# Proactive care of older people undergoing surgery

**DOI:** 10.1007/s40520-017-0879-4

**Published:** 2018-01-04

**Authors:** Judith Partridge, Magda Sbai, Jugdeep Dhesi

**Affiliations:** GSTT, London, UK

**Keywords:** POPS, Proactive care, Older people, Surgery, Comprehensive geriatric methodology

## Abstract

The number of older patients undergoing surgery is increasing due to changing demographics, surgical and anaesthetic advances and shifts in patient expectations of healthcare. The benefits of surgery in older people are well documented and include symptom control and increased life expectancy. However, older surgical patients present not only with the index pathology requiring surgery but with concurrent age related physiological decline, multimorbidity and geriatric syndromes. These additional issues increase the risk of adverse postoperative outcome, in particular of postoperative medical and functional complications. In recent years, there has been recognition of the need for collaborative surgical and geriatric medicine working to address the health care needs of the increasingly complex older surgical population. Guidelines have been published to support clinicians looking after older surgical patients, however, there has been little published on the establishment of such services. In this paper, we describe the evolution of the proactive care of older patients undergoing surgery (POPS) service and how through the use of comprehensive geriatric assessment methodology and intervention throughout the surgical pathway, outcomes for complex older surgical patients can be improved.

## Introduction

Increasing numbers of older patients are undergoing elective and emergency surgery [1]. This is in part due to demographic change but also due to surgical and anaesthetic advances and higher patient and carer expectations regarding healthcare provision in later life. Older patients referred to surgical teams present not only with the index pathology requiring surgery, but also with age-related physiological decline, multimorbidity and geriatric syndromes, often associated with functional limitation. With this pathophysiological profile, it is no surprise that postoperative outcomes are worse in older patients when compared to their younger counterparts. Adverse medical postoperative complications are seen with increasing frequency in older patients in comparison to younger patients, whereas surgical complication rates remain relatively static as age increases. These medical complications include organ specific complications; cardiac (coronary syndromes, atrial fibrillation, heart failure), respiratory (lower respiratory tract infections, exacerbation of COPD) and renal (acute kidney injury). Other postoperative complications which affect disproportionately higher numbers of older patients are postoperative cognitive disorders, including delirium and hospital acquired deconditioning. This is likely to be a consequence of pre-existing geriatric morbidity, for example cognitive impairment or syndromes such as immobility. All of these postoperative complications are directly associated with higher perioperative and longer term mortality rates, as well as higher cost as a consequence of longer length of hospital stay and need for rehabilitation or ongoing care needs [2, 3].

Whilst acknowledging that adverse medical and functional outcomes are prevalent in older patients, it is also important to note the well documented benefits of surgery including symptomatic relief and increased life expectancy. However, balancing the benefits and possible harm from a surgical procedure against alternative intervention or no intervention in a complex older population can be difficult, especially in the context of multiple long-term conditions and limited life expectancy. The balancing of risk–benefit ratio of intervention requires knowledge of the options for surgical and non-surgical interventions, an understanding of the risks and benefits of these proposed interventions, skills in optimisation using evidence based approaches and the ability to apply this knowledge to an individual patient with specific comorbidities, or geriatric syndromes. Furthermore, it requires an understanding of the whole surgical pathway, expertise in shared decision making and an ability to tailor perioperative care to the individual patient.

## The ideal model of care (CGA)

Given the complexities of the new surgical population and their specific health care needs, it is not surprising that the traditional model of surgical care may not be able to deliver the best possible outcomes. This is particularly true when considering the higher levels of multimorbidity (for example, half of all 80-year-old patients in the United Kingdom has three or more coexisting conditions) and geriatric syndromes within this population. In this context, the surgical episode often represents an acute punctuation in the pathway of chronic disease management, with many older patients already being managed on multiple chronic disease pathways of care.

The ideal model of preoperative care for such a complex population should incorporate assessment, optimisation and management of issues pertaining to the surgical episode, whilst taking into context assessment and management of underlying chronic disease and geriatric syndromes. There is an increasing recognition that such an approach can be facilitated through comprehensive geriatric assessment (CGA) and optimisation methodology.

CGA provides a robust evidence based methodology to underpin this ‘ideal model of care’ for the older surgical patient [4]. Familiar to geriatricians, CGA is a multi-domain, usually multidisciplinary assessment of an older patient which aims to identify and manage both existing and newly diagnosed medical, functional and psychosocial issues employing short- and long-term plans for treatment. Within the medical and community setting CGA has been shown to improve the chances that an older person will be alive and living in their own home with cognitive health at 6–18 months following the CGA intervention [5]. Within the surgical setting the evidence base for preoperative CGA and optimising is also growing [6] with emerging literature showing an impact on shorter length of hospital stay attributed to fewer medical complications and on discharge related issues [7] and also an improved 90-day survival [8]. In practical terms, preoperative CGA can be used to systematically and objectively identify and assess severity of both recognised and previously unrecognised pathology and to use evidence based strategies to optimise the patient across medical, social, functional, and psychological domains. This method can help to describe the likely risks related to the surgical episode and prompt the team to use strategies to reduce the incidence and severity of such risks. Furthermore, an awareness of these risks can allow early identification of predictable postoperative complications and ensure a standardised approach to management. Whilst there is clear rationale and an emerging evidence base for using CGA in the perioperative setting, it is considered difficult to implement in routine clinical practice. In this paper, we will describe how CGA has been implemented across surgical specialties at an inner-city teaching hospital in the United Kingdom.

## The evolution of the proactive care of older people undergoing surgery (POPS) service

The POPS team used the Medical Research Council framework for complex interventions [9] to model, design, embed and evaluate the use of CGA in elective surgical settings. Literature searches and policy review confirmed the need for innovative approaches in improving quality care for older complex surgical patients. Exploratory work was undertaken to examine the feasibility of pre-operative comprehensive geriatric assessment (CGA) intervention for older surgical patients. This demonstrated that older patients undergoing elective surgery had high levels of modifiable pre-operative comorbidity, but rarely received geriatric or multidisciplinary team input before surgery. Of those aged 65 and over, 20% had their surgery delayed for preventable medical reasons (for example failure to cease anticoagulation) and there was a high incidence of significant postoperative problems delaying discharge. Opinion was sought from “front line” workers (e.g. surgical nurses, general practitioners) and patients about the potential value of a preoperative intervention service. Following this exploratory work, the POPS pilot was commenced. A questionnaire was posted to patients aged 65 years and over awaiting surgery. These self-completed questionnaires identified potential risk factors known to lead to poor postoperative outcomes. Patients with these risk factors were directly invited to attend a preoperative assessment and optimisation clinic and direct referrals from local consultants and GPs were also encouraged.

At this point, the POPS team comprised a consultant geriatrician (two clinical sessions) and a full-time nurse specialist for older people, occupational therapist, physiotherapist, and social worker. Preoperatively, patients were evaluated using CGA which incorporated validated screening methods or tools (e.g. Hospital Anxiety and Depression Score, Barthel). The multidisciplinary team then preoperatively addressed the identified problems, for example heart failure medication adjusted, anaemia treated and electrolyte imbalance addressed. Education on exercise, nutrition, smoking cessation, and pain management was provided. Therapy input involved anticipation of needs at hospital discharge, and proactive provision of equipment. Postoperatively, the geriatrician and the nurse reviewed patients on the surgical wards, providing hands on care alongside staff education in the early detection and treatment of medical complications (e.g. acute kidney injury, AF, delirium) and multidisciplinary issues (e.g. early mobilisation, pain management, bowel/bladder function, nutrition, and discharge planning). Following discharge, the POPS team provided a follow-up therapy home visit for those with functional difficulties, and outpatient clinic review in those with on-going medical problems. Thereafter, patients were linked with pre-existing services as needed, e.g. falls programmes, continence service, other outpatient services, and the voluntary sector.

The POPS model was evaluated in a pre- and post-study and showed fewer postoperative medical complications [pneumonia 20 vs 4% (*P* = 0.008), delirium 19 vs 6% (*P* = 0.036)], fewer multidisciplinary issues [pressure sores 19 vs 4% (*P* = 0.028), less of a delay to mobilisation 28 vs 9% (*P* = 0.012)] and a reduced length of hospital stay (4.5 days) [10].

More recently the effectiveness of the POPS model was further evaluated in a single site randomised clinical trial undertaken in elective vascular surgical patients (abdominal aneurysm and lower limb revascularisation). This study showed that preoperative CGA and optimisation conducted by a POPS team reduced length of stay by 40% (*P* < 0.001) when compared to standard preoperative processes of care [6]. This was largely attributable to fewer medical complications and streamlined discharge planning. Future work is planned to study the implementation of a POPS type model of care across several centres in the United Kingdom and to facilitate further dissemination.

Based on this published work and data from quality improvement projects the POPS service at the index hospital has continued to evolve over a 12 year period and now provides care for the majority of surgical subspecialties (both elective and emergency) at the host hospital.

The quality improvement approaches used have included;

- Regular multidisciplinary POPS team clinical governance meetings to identify areas for improvement.
- Attendance and presentation at newly established joint surgical and anaesthetic audit meetings.
- Development of cross-speciality clinical guidelines and protocols e.g. pre-operative indications for vena caval filters, perioperative management of diabetes.
- Local quality improvement programmes, e.g. trust wide initiative to promote screening, identification, and management of delirium.
- Engagement with national quality improvement programmes e.g. working collaboratively to address findings from national emergency laparotomy audit (NELA).
- Establishment of a patient public engagement and involvement group to both ensure co-design and co-production of the service.

## The current POPS service

The POPS team now comprises 3 geriatricians, 3 higher registrar trainees, 11 junior doctors, 5 clinical nurse specialists, an occupational therapist, an administrator, and secretary. The service works across two sites covering the majority of surgical sub-specialities and both elective and emergency admissions.

## The clinical service

### Preoperative elective care

When a provisional decision to proceed to surgery is made, all surgical patients are triaged to the appropriate preoperative assessment clinic: either nurse-led preoperative assessment (which is standard care throughout the NHS) or directly to the preoperative POPS assessment and optimisation clinic based on the presence of multimorbidity, geriatric syndromes or concerns about functional status. POPS clinics preoperatively assess 1200–1400 new patients annually. This assessment employs CGA and optimisation utilising multidisciplinary skills. The review aims to:

Assess perioperative risk

- Organ specific risk.
- Overall risk of morbidity and mortality.
- Risk of functional decline/post-operative cognitive disorders.

Medically optimise patients to modify risk

- Optimise known comorbidity.
- Identify and optimise previously unrecognised disease.

Provide functional and psychosocial assessment

- Predict and modify risk of hospital associated deconditioning.
- Predict care needs at hospital discharge.

Promote shared decision making

- Assess capacity.
- Inform discussion of risk–benefit ratio of different treatment options (with surgeon, anaesthetist, and patient).

Provide an individually tailored perioperative management plan

- Covering management of expected complications.
- Proactively communicating with patients, relatives, surgeons, anaesthetists, ward teams, primary care, etc.

### Preoperative emergency care

In emergency patients this process of preoperative assessment and optimisation is tailored to the acuity of surgical intervention. To ensure that emergency surgical patients are directed to the appropriate teams and settings in a timely fashion, a combination of approaches is used. These include early warning scores, frailty assessment scores, delirium risk assessments, and mortality scores. This allows early level 2 and 3 care for the very unwell patient, and proactive involvement from POPS for patients with geriatric syndromes and multi-morbidity.

### Postoperative care (elective and emergency)

The team provides ongoing care for elective patients known to the service and proactively manages emergency patients with a focus on medical complications and functional deterioration. This care is provided through;

- Joint medical–surgical ward rounds.
- Case management on surgical wards.
- Regular medical ward rounds on surgical ward.
- Ward based multidisciplinary team meetings to promote rehabilitation goals and proactive discharge planning (weekly formal meeting or daily board round).
- Proactive communication between hospital staff, patients, and carers.
- Onward referral to appropriate services after hospital discharge.

## Education and training

With the aim of improving care for older surgical patients and developing sustainable services POPS has invested in education and training, both locally and nationally. Local initiatives include;

- Trust-wide nurse education on issues pertinent to older surgical patients (e.g. delirium, falls, medicines management).
- Contribution to nursing masters’ modules within the trust (e.g. vascular clinical nurse specialist).
- Development of POPS clinical nurse specialist role (physical examination skills, nurse prescribing).
- Establishment of junior doctor programme in perioperative medicine for older people.
- Development of year-long specialist registrar programme to train the subspecialist in perioperative medicine for older people.

National initiatives include;

- Establishment of a national 2 day conference attended by geriatricians, anaesthetists, and surgeons covering aspects of perioperative medicine.
- Authorship of a module covering perioperative medicine for older patients as part of an MSc programme.
- Authorship of British Geriatrics Society e-learning module in perioperative medicine for the older patient.
- Development of a curriculum in perioperative medicine for the older patient for geriatric medicine specialist registrar trainees (endorsed by the Royal College of Physicians/British Geriatrics Society).

## The national uptake of POPS models of care in the UK

The emerging evidence base for POPS type interventions (preoperative CGA and optimisation) coupled with an increasing recognition of the need for collaborative working, has resulted in several high profile national UK reports which advocate the involvement of geriatricians in complex older surgical patients. These include:

- National Confidential Enquiry into Patient Outcome and Death, ‘An age old problem’ 2010 [11].
- Royal College of Surgeons and Age UK, ‘Access all Ages 1&2’, 2013, 2015 [12, 13].
- Royal College Anaesthetists, Perioperative Medicine Programme 2015 [14].

Despite this, widespread adoption of POPS models has not yet occurred as seen in a survey of UK hospitals. This showed that in 2014, of 161 acute centres only three had adopted POPS input throughout the pathway with a further 28 providing either pre- or postoperative care [15]. However, since this study an encouraging number of new services have been established with promising results in terms of quality of care and improved postoperative outcomes. An updated national survey is underway and will be used to describe and address the barriers and challenges faced by innovators in this field.

## Conclusions

As evidence emerges that services such as POPS should become part of routine clinical care for older patients undergoing surgery, a number of challenges become apparent. These include discussion about which speciality (anaesthetics, single organ specialities or geriatricians) are best placed to offer such services and indeed which discipline can and should deliver them (nursing, medical or therapies). This has clear implications for workforce planning especially in view of the limited numbers of geriatricians available to deliver not only core geriatric medicine services but also these more innovative models of care. Furthermore, the emerging speciality of perioperative medicine requires specialist education and training to understand the complexities of the older patient in the context of the surgical episode. Whilst these issues require re-allocation of resources, both financial and workforce, the evolving picture of POPS service development in the UK demonstrates that innovation in this setting is possible and can be beneficial in terms of quality of care provided to older surgical patients.

## References

[1] Etzioni DA, Liu JH, Maggard MA, Ko CY (2003). The aging population and its impact on the surgery workforce. Ann Surg.

[2] Hamel MB, Henderson WG, Khuri SF, Daley J (2005). Surgical outcomes for patients aged 80 and older: morbidity and mortality from major noncardiac surgery. J Am Geriatr Soc.

[3] Polanczyk CA, Marcantonio E, Goldman L, Rohde LE, Orav J, Mangione CM (2001). Impact of age on perioperative complications and length of stay in patients undergoing noncardiac surgery. Ann Intern Med.

[4] Stuck AE, Siu AL, Wieland GD, Adams J, Rubenstein LZ (1993). Comprehensive geriatric assessment: a meta-analysis of controlled trials. Lancet.

[5] Ellis G, Gardner M, Tsiachristas A, Langhorne P, Burke O, Harwood RH, Conroy SP, Kircher T, Somme D, Saltvedt I, Wald H, O’Neill D, Robinson D, Shepperd S (2017). Comprehensive geriatric assessment for older adults admitted to hospital. Cochrane Database Syst Rev.

[6] Partridge JSL, Harari D, Martin FC, Dhesi JK (2014). The impact of pre-operative comprehensive geriatric assessment on postoperative outcomes in older patients undergoing scheduled surgery: a systematic review. Anaesthesia.

[7] Partridge JSL, Harari D, Martin FC, Peacock JL, Bell R, Mohammed A, Dhesi JK (2017). Randomized clinical trial of comprehensive geriatric assessment and optimization in vascular surgery. Br J Surg.

[8] McIsaac DI, Huang A, Wong CA, Wijeysundera DN, Bryson GL, van Walraven C (2017). Effect of preoperative geriatric evaluation on outcomes after elective surgery: a population-based study. J Am Geriatr Soc.

[9] Craig P, Dieppe P, Macintyre S, Michie S, Nazareth I, Petticrew M (2008). Developing and evaluating complex interventions: the new Medical Research Council guidance. BMJ.

[10] Harari D, Hopper A, Dhesi J, Babic-Illman G, Lockwood L, Martin F (2007). Proactive care of older people undergoing surgery (‘POPS’): designing, embedding, evaluating and funding a comprehensive geriatric assessment service for older elective surgical patients. Age Ageing.

[11] Wilkinson K, Martin IC, Gough MJ et al (2010). An age-old problem: care of older people undergoing surgery. NCEPOD, London. http://www.ncepod.org.uk/2010report3/downloads/EESE_fullReport.pdf. Accessed 14 Oct 2017

[12] The Royal College of Surgeons of England (2012) Access all ages: assessing the impact of ages on access to surgical treatment. RCSENG, London. https://www.rcseng.ac.uk/library-and-publications/college-publications/docs/access-all-ages/. Accessed 14 Oct 2017

[13] The Royal College of Surgeons of England (2014). Access all ages 2. RCSENG, London. https://www.rcseng.ac.uk/library-and-publications/college-publications/docs/access-all-ages-2/. Accessed 14 Oct 2017

[14] The Royal College of Anaesthetists (2014) Perioperative medicine the pathway to better surgical care. RcoA, London. https://www.rcoa.ac.uk/periopmed/vision-document. Accessed 14 Oct 2017

[15] Partridge JSL, Collingridge G, Gordon AL, Martin FC, Harari D, Dhesi JK (2014). Where are we in perioperative medicine for older surgical patients? A UK survey of geriatric medicine delivered services in surgery. Age Ageing.

